# Enhanced Associations With Actions of the Artist Influence Gaze Behaviour

**DOI:** 10.1177/2041669520911059

**Published:** 2020-03-12

**Authors:** Louis Williams, Eugene McSorley, Rachel McCloy

**Affiliations:** School of Psychology and Clinical Language Sciences, University of Reading

**Keywords:** gaze behaviour, congruency, artistic actions

## Abstract

The aesthetic experience of the perceiver of art has been suggested to relate to the art-making process of the artist. The artist’s gestures during the creation process have been stated to influence the perceiver’s art-viewing experience. However, limited studies explore the *art-viewing* experience in relation to the creative process of the artist. We introduced eye-tracking measures to further establish how congruent actions with the artist influence perceiver’s gaze behaviour. Experiments 1 and 2 showed that simultaneous congruent and incongruent actions do not influence gaze behaviour. However, brushstroke paintings were found to be more pleasing than pointillism paintings. In Experiment 3, participants were trained to associate painting actions with hand primes to enhance visuomotor and visuovisual associations with the artist’s actions. A greater amount of time was spent fixating brushstroke paintings when presented with a congruent prime compared with an incongruent prime, and fewer fixations were made to these styles of paintings when presented with an incongruent prime. The results suggest that explicit links that allow perceivers to resonate with the artist’s actions lead to greater exploration of preferred artwork styles.

There has been a great deal of research that examines what features of artworks affect the aesthetic experience, and what aspects appear to play an important role in them being deemed aesthetically pleasing. However, it has been argued that to better understand the aesthetic experience of art, research must consider the artist behind the artwork, their motivations, decisions, and actions (Dutton, 2009; Tinio, 2013; Williams et al., 2018). A link between the artist’s experience while producing art with the aesthetic experience of the perceiver has been made explicit in the Mirror Model of Art put forward by Tinio (2013). Here, a direct link is drawn between three general stages common to many models of aesthetic experience (from low-level visual processing to higher level cognitive processing before a final aesthetic judgement and emotional response; see Chatterjee, 2011; Leder et al., 2004) and stages identified in the act of art creation and production (from ideas and conception through development to the finishing touches; see Mace & Ward, 2002). The suggestion is that the direction of travel through these stages runs counter to each other. Therefore, the artist first has several ideas, develops and isolates one, and then finishes the work. The perceiver’s aesthetic experience arises from a reverse journey through this process. First, there is an initial survey of the work driven by low-level visual processes dictated largely by the work’s finishing touches. Then, further exploration of the work takes place as higher level cognitive processes come into play. Finally, emotional reactions and aesthetic judgements are reached driven by the artist’s underlying idea (Tinio, 2013).

Aspects of the art-making process are therefore suggested to influence the aesthetic experience of the perceiver, and there is some evidence supporting this impact of the creator. The perceiver’s knowledge of the artist behind the artwork, having a view into their art-making process, and simulating such behaviours, has been found to influence the aesthetic experience (Chamberlain et al., 2018; Leder et al., 2012).

## The Artist Behind the Artwork: Aesthetic Judgements

Details such as the title and information about the artwork, the identity of, or information about the artist and art style, have been found to impact aesthetic experiences (Millis, 2001; Temme, 1992). Naïve viewers who attended lectures about art styles (abstract paintings) and gained knowledge about these methods were found to have higher aesthetic ratings for abstract paintings than those who received a lecture on Renaissance art, or no lecture at all (Stojilović & Marković, 2014). Chamberlain et al. (2018) showed how computer-generated artworks are rated as less pleasing than man-made pieces. However, when the production process of computer-generated art was observed during aesthetic evaluation, then higher aesthetic ratings were made. Furthermore, Sbriscia-Fioretti et al. (2013) found that participants perceived more movements in real artworks in comparison with modified computerised versions, with higher aesthetic ratings also being assigned to these artworks. They state that the dynamics of the artworks where the brushstrokes made by the artist can be perceived influenced these results, suggesting the importance of the actions of the artist behind the artwork.

Simply observing objects that represent action have been found to lead to covert simulations allowing an object to be easily processed, ultimately influencing the aesthetic experience (Ping et al., 2009). Mimicry of actions and gestures are therefore suggested to influence aesthetic judgements alone. Bertamini et al. (2013) found that congruent postures between participants and an observed 3-D model increased perceived attractiveness despite the use of static images where no active movement could be observed. Here, participants were also unaware that they were creating congruent postures.

Researchers have begun to further explore relationships between congruent actions and aesthetic judgements within the realm of art-making. Leder et al. (2012) conducted a study where they examined how creating simultaneous actions (while hands were hidden) influenced perceiver’s aesthetic judgements of artworks, particularly if these actions were congruent or incongruent to the original actions of the artist. They found that congruent actions with the artist positively impacted the perceiver’s liking of art. However, replications of this study have failed to find an impact of congruent action on the perceiver’s experience (McLean et al., 2015; Woltin & Guinote, 2015). Though, McLean et al. (2015) found an impact of congruent action on aesthetic ratings when using alternative stimuli and when explicitly informing participants about the relationships between the actions portrayed in the artwork and those being simultaneously made by the participant. This therefore questions whether the simple presentation of static stimuli allows for the gestures of the artist to impact the aesthetic experience of the perceiver. It may be the case that simultaneous actions alone are not enough to examine the relationships between artist and perceiver, providing a more obvious connection between the participant’s action and the artwork may be important.

This suggestion is supported by the work of Ticini et al. (2014). They introduced a visuomotor training phase. In this training phase, participants were given a paintbrush and paint and carried out stippling actions, stroking actions, or no action at all when presented with a photograph representing a congruent hand grip. Notably, in this phase, their hands were visible, and they were instructed to *paint dots* or *paint strokes*, thus making the link between action and artwork very explicit. In the testing phase, prior to observing pointillism paintings, a priming hand grip photograph was also presented where a pretrained *action* photograph was shown, again enhancing the visuovisual associations. Perceivers rated pointillism paintings higher on a liking scale when presented with the congruent hand prime.

Regardless of an implicit or more explicit link being made between the artist and the perceiver, whether through providing perceivers with information about the artist, allowing them to view the art-making process, or through mimicry of actions, it is apparent that the artist behind the artwork influences perceiver’s aesthetic judgements. Research has also begun to further explore how the artist behind the artwork influences gaze behaviour.

## The Artist Behind the Artwork: Gaze Behaviour

Eye movements have been found to provide a wider and more nuanced picture of the way in which the perceiver examines an artwork while experiencing and forming an aesthetic relationship with it. Therefore, eye movements provide an insight into the impact of the structure and content of stimuli on the aesthetic experience (Locher et al., 2007). Gaze can be particularly well suited to elucidate the formation of aesthetic judgements including early responses. It has been shown that early reactions to pictures begin within the first 2 seconds of viewing; experiences here are influenced more by the low-level features, the pictorial elements, the style, and form (Locher, 2006, 2015). Williams et al. (2018) examined gaze behaviour to pairs of geometric stimuli that differed in shape and complexity. Artists and nonartists were found to fixate first, for longer and more often on stimuli rated to be more pleasing, supporting conclusions that eye movements provide further insight into the formation of aesthetic judgements. However, in this study, there were no apparent differences in the creative process behind the stimuli.

Artists are suggested to particularly enhance low-level visual attributes of their work including motion, luminance, and colours to impact their perceiver’s response (Chatterjee, 2011). Massaro et al. (2012) highlights how eye-tracking techniques can provide a further understanding of an aesthetic experience by investigating the impact of low-level features such as colour and dynamism, as well as top-down processes represented by the task at hand and content of the stimuli. Brinkmann et al. (2019) investigated the impact of dynamic artistic actions on gaze behaviour. They found the number of fixations made, saccade velocity, and aesthetic judgements to increase due to individual perceptions of dynamic movement within the artwork. This provides further support for the importance of exploring gaze behaviour as we begin to consider the process of the artist and how such dynamic movements influence perceiver’s gaze.

Gaze behaviour and aesthetic judgements have been found to differ due to the background of the artist and perceivers gaining knowledge about the creative process (Alvarez et al., 2015; Ishiguro et al., 2016). Alvarez et al. (2015) explored how pairing artworks that differed due to the type of artist behind the artwork (artist, child, or animal) influenced aesthetic judgements in terms of quality and preference but also eye movement behaviour. They found that despite participants not being explicitly informed about the creator, artworks created by artists were assigned higher quality ratings, but no preferential differences were found. While making quality ratings, artist’s productions were fixated on more and evoked greater pupil dilation. While making preference judgements, artist’s productions also evoked greater pupil dilation. The researchers emphasise the importance of examining eye movements that provide an insight into how perceivers implicitly explore the structure of an artwork, and how gaze behaviour towards artworks is influenced by the artist behind the artwork. Ishiguro et al. (2016) aimed to further understand how providing an in-depth experience of the technical features and the creative process of the artist behind the artwork, in this instance, photography, influenced gaze behaviour during art observation. Eye movement data demonstrated that after completing an artistic course designed to inform participants of the artistic process of photo creation, perceiver’s gaze behaviour changed. Perceiver’s average fixation duration for photographs that contained recognisable features, such as objects or humans, reduced after training, and an increase in the global number of saccades when viewing classic photographs was found, regardless of containing recognisable features. The results suggest more active exploration once having a greater understanding of the creative process. This raises further questions of how gaze behaviour is influenced by stimuli and tasks that allow the perceiver to resonate with the artist behind the artwork.

## The Present Study

As gaze behaviour has been suggested to be driven by aesthetic preferences but also influenced by the creator behind the artwork and knowledge of their creative process (Alvarez et al., 2015; Holmes & Zanker, 2012; Ishiguro et al., 2016; Williams et al., 2018), we aim to further examine gaze behaviour towards artworks when perceivers are involved in tasks that allow them to resonate with the actions of the artist behind the artworks observed. Leder et al. (2012), McLean et al. (2015), and Ticini et al. (2014) all show that aesthetic judgements can be influenced due to creating congruent actions with the artist in stipple or stroke form. Here, we further explore congruent actions and its impact on perceiver’s gaze behaviour.

Pleasingness ratings and eye movements were recorded. We present three experiments that consider the responses of both artists (only in Experiments 1 and 2) and nonartists. In Experiment 1, we use geometric stimuli differing in style (stippling and stroking) that clearly shows the differing actions during creation. In Experiment 2, we conduct a similar method; however, we present artworks (pointillism and brushstroke paintings), thus displaying stimuli made by artists who use either brushstroke or stippling actions. Finally, in Experiment 3, we use a training paradigm of artistic actions and incorporate a priming task providing a more explicit link with the motoric actions of the artists behind the artworks. Data for all three studies, stimuli, and supplementary materials are freely available on Open Science Framework (doi: 10.17605/OSF.IO/87E42). We are happy for researchers to reanalyse our data or reuse our stimuli for any purpose.

## Experiment 1

Research has explored how connections with the artist behind the artwork, particularly due to performing congruent actions, influence aesthetic judgements (Leder et al., 2012; McLean et al., 2015; Ticini et al., 2014). Researchers have also demonstrated how providing a window into the art-making process of the artist influences gaze behaviour (Ishiguro et al., 2016). However, further research is required to understand whether perceiver’s gaze behaviour is influenced by resonating with the artist, particularly regarding the artistic actions and gestures that are produced. This current study examines this relationship between the artist and perceiver considering expertise and two differing styles of drawing (stippling and stroking) using geometric stimuli where the actions of the artist are obvious to the viewer. McLean et al. (2015) found a congruency effect only when using stimuli that clearly portrayed the actions of the creator and when participants were made explicitly aware of these connections. In this experiment, we use white geometric drawings that differ in symmetry that were created by clear strokes (stroking action) or clear dots (stippling action). This removes the potential influences of familiarity, content, and colour and allows the techniques of the artist to be clearly displayed. Using abstract stimuli is ideal as more attention can be made to the low-level features due to the lack of content within the stimulus (Cupchik et al., 2009; McLean et al., 2015; Nadal, 2013; Stojilović & Marković, 2014).

We hypothesise that aesthetic ratings for congruent images will be higher than those for incongruent images and those in the control group who do not make any simultaneous actions. Those in the “stroking” condition (in which line strokes were created by pencil) will prefer stroke stimuli. Those in the “stippling” condition (in which dotted marks were created by pencil) will prefer stipple stimuli. We also hypothesise stronger effects of congruency to be found with artists. They have more experience with artistic actions and are suggested to consider the creative process more so during art observation (Chatterjee & Vartanian, 2016; Ishiguro et al., 2016; Pitman & Hirzy, 2010).

When we consider the perceiver, it is suggested that the low-level features of artworks including the styles and techniques of the artist have an impact on perceptual processes that can be detected in gaze behaviour (Locher, 2006, 2015). It is further suggested that the creator behind the artwork, and more specifically knowledge of their creative process, influences eye movement behaviour (Alvarez et al., 2015; Ishiguro et al., 2016). We hypothesise that gaze (first fixation, first saccade latency, fixation duration, and fixation count) will be directed to and greatly influenced by congruency. Those in the stroking condition will fixate on stroke stimuli, and those in the stippling condition will fixate on stipple stimuli. Again, we hypothesise a stronger effect of congruency with artists.

### Method

#### Participants

A total of 81 participants took part in this study; 48 psychology students were recruited from the University of Reading and regarded as nonartists (27 females, 21 males: ages 19–50), and 33 student artists (25 females, 8 males: ages 20–49) were recruited from the fine art department at the University of Reading and regarded as artist’s using a background questionnaire. The questionnaire requested the participant to provide the number of years of formal art training (A-level qualification and beyond) and number of years of art experience they had received. Years of formal art training for the artists ranged from 5 to 7 years (*M *=* *5.6 years) who had from 5 to 18 years (*M *=* *6.5 years) of art experience. The nonartists had less than 2 years (*M *=* *0.1 years) of training and no years of art experience. All participants had normal or corrected-to-normal vision and completed each stage of the study.

#### Materials

**Figure 1.**
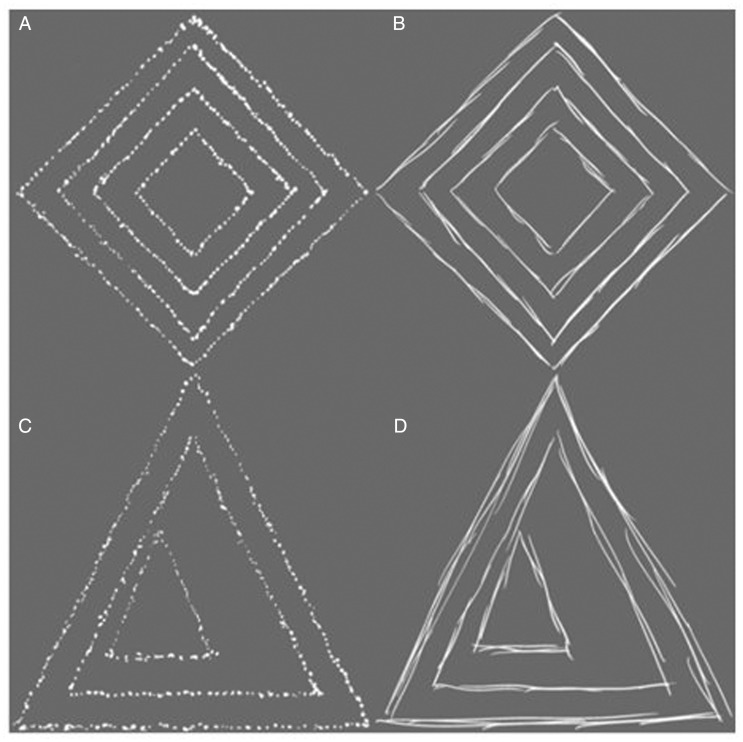
Examples of stimuli used (four out of eight) from the four pairs: A: stipple-symmetrical (Pair 1). B: stroke-symmetrical (Pair 2). C: stipple-asymmetrical (Pair 3). D: stroke-asymmetrical (Pair 4).

The stimuli included eight grayscale geometric shapes created on a Microsoft Surface computer tablet (see Figure 1). The aforementioned stimuli were drawn using stroking and stippling action allowing for the actions of participants to match those made when creating the stimuli, thus enhancing the visual associations between the images and the actions being produced (McLean et al., 2015). These images were grouped into four pairs based on their symmetry and style, with each pair containing two images (stipple-symmetrical, stroke-symmetrical, stipple-asymmetrical, and stroke-asymmetrical).

**Figure 2.**
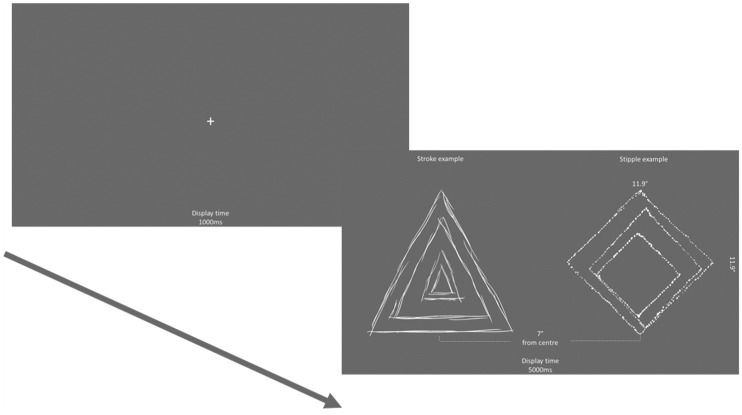
Schematic trial example.

A 7-point scale was used to gather aesthetic ratings (1: *very displeasing* to 7: *very pleasing*). The participants made these responses verbally. A verbal response was used here due to consideration of methods used in previous studies (Furman & Duke, 1988; Martindale et al., 1990). Leder et al. (2012) recorded liking ratings using a keyboard press, and McLean et al. (2015) requested participants to tick relevant scores on paper. These response actions may interfere with the stroking and stippling actions that participants are simultaneously making, for example, the rhythm and action of these may be muted while making judgements, and the particular action to respond may interfere with the painting action being produced. Therefore, verbal response enables the actions to continue without a pause and does not bias the stippling or stroking actions.

A debrief questionnaire was also given at the end of the study to examine if participants were aware of the connection between the actions they made and the actions portrayed in the images observed. They were also asked if they felt that this relationship influenced their aesthetic ratings.

#### Apparatus

Stimuli were presented on a 21″ colour desktop PC running Experiment Builder (SR Research Ltd, Ottawa, Ontario, Canada). The distance between the monitor and participant was 57 cm. Stimulus width and height subtended 11.9° of visual angle. Two images were presented per trial centred 7° to the left and right of the vertical meridian and centred on the horizontal meridian. All images were presented on a grey background. Eye movements of the right eye were recorded using an EyeLink II tracker with a sampling rate of 500 Hz. A chin rest was used, and participants were placed in a set position and requested not to move during the study. Calibration was maintained for each trial using a standard drift correct procedure between each trial that corrected fixation errors due to small movements in camera alignment (e.g., caused by head band slippage).

#### Design and Procedure

Participants were randomly assigned to one of three action conditions. In the stippling condition (nonartist: *n *=* *16, artist: *n *=* *14), participants were requested to tap a pencil on paper at their own pace. In the stroking condition (nonartist: *n *=* *16, artist: *n *=* *11), participants were asked to draw straight lines at their own pace; for both conditions, participants’ hand movements could not be observed. The control group (nonartist: *n *=* *16, artist: *n *=* *8) were not requested to make any actions. Thus, there were three independent variables: image style (brushstroke and pointillism); action type (control, stipple, and stroke); and expertise (nonartist and artist).

##### Aesthetic Rating Task

This task required participants to rate (verbally) how visually pleasing they found each image (1–7). Participants were randomly required to complete this task either before or after the eye-tracking task to control for any potential order effects. All images were presented for 5,000 ms prior to making an aesthetic judgement.

##### Eye-Tracking Task

For the free-view eye-tracking task, participants were simply informed that they would be presented with pairs of images that they could freely view. Participants’ eye movements were analysed while they were presented with two images for 5,000 ms. A fixation cross was displayed between each trial for 1,000 ms, and a drift correction was applied. A total of 32 trials were completed at random; all stipple images were presented alongside all stroke images. These pairings were shown twice allowing each image in a pair to be presented on both sides of the screen; no further information was provided for this task (see Figure 2).

#### Eye-Tracking Analyses

A variety of gaze metrics were used including first fixation direction (to the left or right stimulus), first saccade latency (the response time from stimuli onset to the start of the first saccadic eye movement response), total fixation duration (the total amount of time spent on each stimulus), and number of fixations (the total number of fixations on each stimulus). Such fixation metrics are useful to understand aesthetic preferences (Holmes & Zanker, 2012). Fixations were classified as such if they exceeded 100 ms and classified as being towards the left or right image simply if they fell on the left or right hand side of the display. Trials were categorised based on fixation metrics towards stipple or stroke images to understand if gaze was influenced by image style and congruent action.

#### Ethics

This study (including all three reported experiments) was approved by the University of Reading Ethics Committee in The School of Psychology and Clinical Language Sciences.

### Results

#### Effect of Congruent Action on Aesthetic Ratings

**Figure 3.**
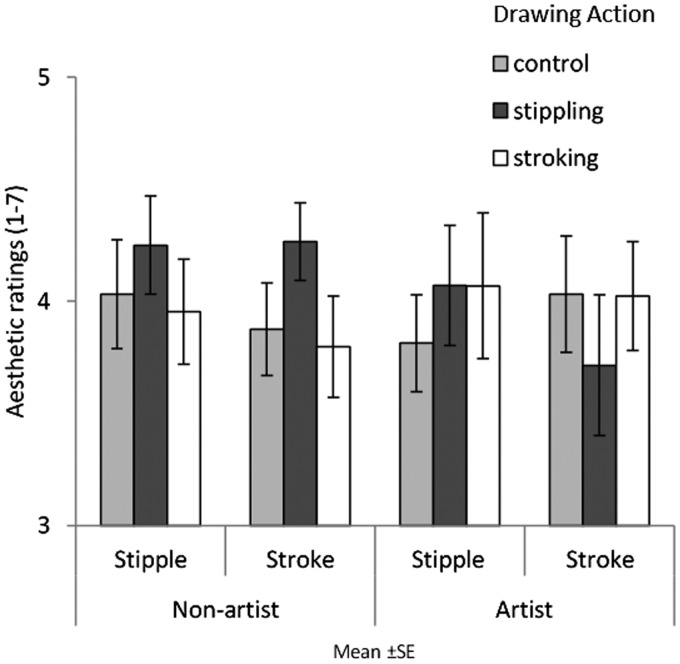
Mean aesthetic ratings of pointillism and brushstroke paintings for both artists and nonartists in the three assigned conditions (control, stippling, and stroking). Error bars show between-group standard error.

Figure 3 shows aesthetic ratings for image style as a function of action conditions and expertise. A three-way analysis of variance (ANOVA) with image style, action type, and expertise was conducted. No main effect of image style was found, *F*(1, 75) = 0.398, mean square error (*MSE*)* *=* *0.694, *p *=* *.530, ${\text{η}}^{\text{2}}$ = .005; no main effect of action type was found, *F*(1, 75) = 0.198, *MSE *=* *0.948, *p *=* *.821, ${\text{η}}^{\text{2}}$ = .005; and no main effect of expertise was found, *F*(1, 75) = 0.032, *MSE *=* *0.948, *p *=* *.858, ${\text{η}}^{\text{2}}$ < .001. No significant interactions were found between image style and action type, *F*(2, 75) = 0.156, *MSE *=* *0.694, *p *=* *.856, ${\text{η}}^{\text{2}}$ = .004; image style and expertise, *F*(1, 75) = 0.010, *MSE *=* *0.694, *p *=* *.920, ${\text{η}}^{\text{2}}$ < .001; action type and expertise, *F*(2, 75) = 1.258, *MSE *=* *0.948, *p *=* *.290, ${\text{η}}^{\text{2}}$ = .032; or image style, action type, and expertise, *F*(2, 75) = 0.629, *MSE *=* *0.694, *p *=* *.536, ${\text{η}}^{\text{2}}$ = .016.

#### Effect of Congruent Action on Gaze Behaviour

**Figure 4.**
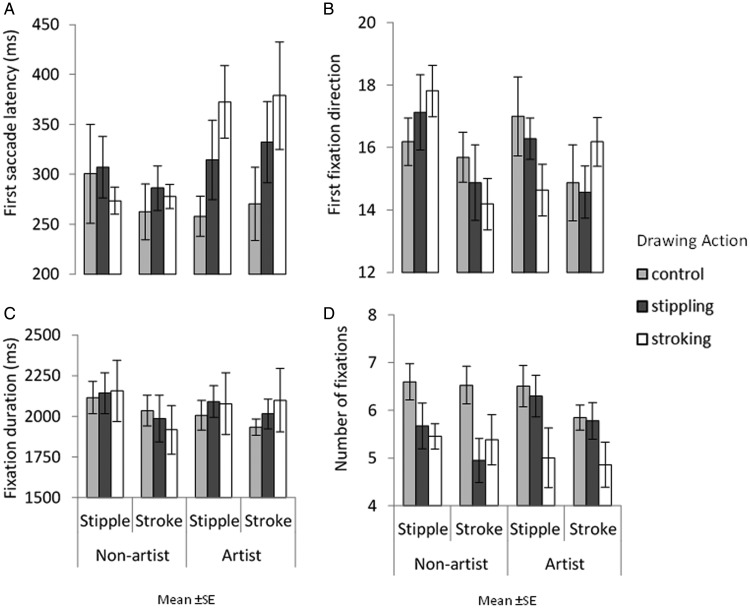
Upper row shows first saccade response: the mean latency of the response in milliseconds (A) and its direction, which is collapsed across expertise and action condition (B). Lower row shows overall fixation behaviour: mean total fixation duration in milliseconds (C) and the mean number of fixations, which is collapsed across image style and expertise (D). Error bars show between-group standard error.

Figure 4 shows gaze behaviour elicited during the eye-tracking task. A series of three-way ANOVAs were conducted for first saccade latency, first fixation direction, fixation duration, and fixation count with image style, action type, and expertise as factors.

**Figure 5.**
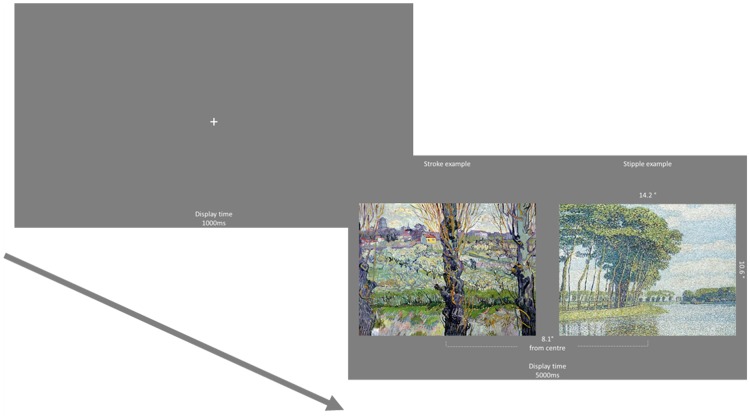
Schematic trial example.

No main effect of image style on first saccade latency was found (Figure 4A), *F*(1, 75) = 0.092, *MSE *=* *4019.539, *p *=* *.762, ${\text{η}}^{\text{2}}$ < .001; no main effect of expertise was found, *F*(1, 75) = 1.837, *MSE *=* *27370.99, *p *=* *.179, ${\text{η}}^{\text{2}}$ = .024; and no main effect of action type was found, *F*(2, 75) = 1.245, *MSE *=* *27370.99, *p *=* *.294, ${\text{η}}^{\text{2}}$ = .032. No significant interactions were found between image style and action type, *F*(2, 75) = 0.242, *MSE *=* *4019.539, *p *=* *.786, ${\text{η}}^{\text{2}}$ = .006; image style and expertise, *F*(1, 75) = 2.174, *MSE *=* *4019.539, *p *=* *.145, ${\text{η}}^{\text{2}}$ = .028; action type and expertise, *F*(2, 75) = 1.540, *MSE = *27370.99, *p *=* *.221, ${\text{η}}^{\text{2}}$ = .039; or image style, action type, and expertise, *F*(2, 75) = 0.490, *MSE *=* *4019.539, *p *=* *.614, ${\text{η}}^{\text{2}}$ = .013.

First fixation directions (Figure 4B) show no main effect of image style, *F*(1, 75) = 3.251, *MSE *=* *24.341, *p *=* *.075, ${\text{η}}^{\text{2}}$ = .042. A main effect of expertise was found, *F*(1, 75) = 17.792, *MSE *=* *0.320, *p *<* *.001, ${\text{η}}^{\text{2}}$ = .192, but a difference in first fixation directions cannot be explained by expertise but slight variations in the number of mean trials reported. No main effect of action type was found, *F*(2, 75) = 2.509, *MSE *=* *0.320, *p *=* *.088, ${\text{η}}^{\text{2}}$ = .063. A significant interaction between action type and expertise was found, *F*(2, 75) = 4.072, *MSE = *0.320, *p *=* *.* *021, ${\text{η}}^{\text{2}}$ = .098. However, again this is due to a variation in the mean number of trials reported across expertise and action type conditions. No significant interactions were found between image style and action type, *F*(2, 75) = 0.135, *MSE *=* *24.341, *p *=* *.* *874, ${\text{η}}^{\text{2}}$ =.004; image style and expertise, *F*(1, 75) = 0.721, *MSE *=* *24.341, *p *=* *.399, ${\text{η}}^{\text{2}}$ = .010; or image style, action type, and expertise, *F*(2, 75) = 1.508, *MSE *=* *24.341, *p *=* *.228, ${\text{η}}^{\text{2}}$ = .039.

Fixation durations (Figure 4C) show no main effect of image style, *F*(1, 75) = 1.046, *MSE *=* *508914.9, *p *=* *.310, ${\text{η}}^{\text{2}}$ = .014; no main effect of expertise was found, *F*(2, 75) = 0.845, *MSE *=* *21360.37, *p *=* *.361, ${\text{η}}^{\text{2}}$ = .011; and no main effect of action type was found, *F*(1, 75) = 1.088, *MSE *=* *21360.37, *p *=* *.342, ${\text{η}}^{\text{2}}$ = .028. A significant interaction between action type and expertise was found, *F*(2, 75) = 3.487, *MSE = *21360.37, *p *=* *.036, ${\text{η}}^{\text{2}}$ = .085. Pairwise comparison reveal that for those in the control condition, nonartists mean fixation duration to an image was greater (*M *=* *2074.776) than artists (*M *=* *1968.091). No significant interactions were found between image style and action type, *F*(2, 75) = 0.003, *MSE *=* *508914.9, *p *=* *.997, ${\text{η}}^{\text{2}}$ < .001; image style and expertise, *F*(1, 75) = 0.118, *MSE *=* *21360.37, *p *=* *.732, ${\text{η}}^{\text{2}}$ = .002; or image style, action type, and expertise, *F*(2, 75) = 0.190, *MSE *=* *508914.9, *p *=* *.827, ${\text{η}}^{\text{2}}$ = .005.

For fixation count, no main effect of image style was found (Figure 4D), *F*(1, 75) = 2.386, *MSE *=* *2.091, *p *=* *.127, ${\text{η}}^{\text{2}}$ = .031. There was a main effect of action type on fixation count, *F*(2, 75) = 5.115, *MSE *=* *3.263, *p *=* *.008, ${\text{η}}^{\text{2}}$ = .120, with participants in the stroking condition making fewer fixations to an image (*M *=* *5.18) than those in the control condition (*M *=* *6.37, *p *=* *.006). No main effect of expertise was found, *F*(1, 75) = 0.027, *MSE *=* *3.263, *p *=* *.870, ${\text{η}}^{\text{2}}$ < .001. No significant interactions were found between image style and action type, *F*(2, 75) = 0.445, *MSE *=* *2.091, *p *=* *.643, ${\text{η}}^{\text{2}}$ = .012; image style and expertise, *F*(1, 75) = 0.109, *MSE *=* *2.091, *p *=* *.742, ${\text{η}}^{\text{2}}$ < .001; action type and expertise, *F*(2, 75) = 1.932, *MSE = *3.263, *p *=* *.152, ${\text{η}}^{\text{2}}$ = .049; or image style, action type, and expertise, *F*(2, 75) = 0.238, *MSE *=* *2.091, *p *=* *.788, ${\text{η}}^{\text{2}}$ = .006.

### Discussion

It has been suggested that the art-making process of the artist has a direct impact on the perceiver’s art-viewing experience (Tinio, 2013). We investigated this claim further by exploring perceiver’s gaze behaviour while creating simultaneous congruent and incongruent actions with the artist. Regarding aesthetic judgements that are made, Leder et al. (2012) found that pointillism paintings were liked more when perceivers made stippling motions, and brushstroke paintings were like more when perceivers made stroking actions. No effect of congruency was found in the current study for both pleasingness ratings and gaze behaviour regardless of level of artistic expertise. We found a general effect of simultaneous action on gaze behaviour; those creating stroking actions made fewer fixations in comparison with those in the control condition. However, results do not support the hypotheses of Experiment 1. This is the case despite the use of geometric drawings that served to enhance the motor associations between one’s own action and the observed images (McLean et al., 2015). It is important to note that some participants were exposed to images during the eye-tracking task prior to evaluations. However, the order of tasks was counterbalanced, and this did not influence aesthetic ratings. Postexperiment interviews revealed that artists were more aware of the actions involved in producing the artworks and how it was reflected in their own actions. Despite this, both artists and nonartists did not feel that creating simultaneous actions influenced their aesthetic judgements, and our findings support this.

## Experiment 2

Experiment 1 showed no evidence to support the suggestion that gaze behaviour is influenced by mimicking the actions of the artist; furthermore, aesthetic ratings were not found to be influenced by such actions. One reason for not finding a congruency effect here may be due to our use of geometric drawings. It is notable that Leder et al. (2012), McLean et al. (2015), and Woltin and Guinote (2015) used paintings, artworks that had been created for exhibition, although McLean et al. (2015) only found a congruency effect using participants’ own drawing productions as stimuli. One clear advantage of using such stimuli is that there are fewer potential confounding factors associated with the content, familiarity, and style of the artworks and the technical expertise of the artists that may play a role in the formation of aesthetic liking. On the other hand, one potential problem is that such stimuli may not be classed as art, or works created by an artist, and thus the connection between congruent actions and artwork style may be weaker here. Furthermore, Chatterjee (2011) suggests that the visual properties of artworks can attract viewer’s attention more than simple objects; thus, artworks may have a greater effect on the perceiver’s eye movements that allows a congruency effect to manifest itself. To examine this, we replicate the methods used in Experiment 1 using artworks as stimuli rather than drawings of geometric shapes. As we introduce existing artworks into this study, we also take note of participants’ familiarity of artworks.

We examine if diverse actions influence the perceiver’s experience when they are simultaneously acting in a congruent or incongruent manner (actions of stippling or stroking) using both subjective aesthetic ratings and eye-tracking methods. We hypothesise that aesthetic ratings for congruent artworks will be higher than those for incongruent artworks and those in the control group who do not make any simultaneous actions. We hypothesise that gaze (first fixation, first saccade latency, fixation duration, and fixation count) will be influenced by congruent stimuli. Thus, greater fixation will be towards congruent artworks compared with incongruent artworks. Although we found no difference between artists and nonartists in Experiment 1, we hypothesise that using our new set of stimuli (brushstroke and pointillism paintings), stronger congruency effects will be observed for artists.

### Method

#### Participants

A total of 72 participants, who were not involved in Experiment 1, took part in this study. Of these, 48 were psychology students recruited from the University of Reading who were regarded as nonartists (27 females, 21 males: range 18–26), and 24 student artists (21 females, 3 males: ranger 20–49) were recruited from the fine art department at the University of Reading. Artist/nonartist status was ascertained using the same questionnaire to Experiment 1. Years of formal art training and art experience for the artists ranged from 5 to 9 years (*M *=* *5.4 years). The nonartists had less than 2 years (*M *=* *0.15 years) of training and less than 2 years (*M *=* *0.12 years) of art experience. All participants had normal or corrected-to-normal vision and completed each stage of the study.

#### Materials

Twelve paintings were used: 6 neoimpressionist pointillism paintings and 6 postimpressionist brushstroke paintings, 10 of which were used by Leder et al. (2012) and McLean et al. (2015; see supplemental materials for list of works). A 7-point scale was used to gather aesthetic ratings (1: *very displeasing* to 7: *very pleasing*). At the end of the study, participants were asked to rate how familiar they were with each painting (1: *very unfamiliar* to 7: *very familiar*); again, all responses were given verbally. A debrief questionnaire form similar to Experiment 1 was also provided.

#### Apparatus

Stimuli were presented on a 21″ colour desktop PC that had a refresh rate of 75 Hz running Experiment Builder (SR Research Ltd.). The distance between the monitor and participant was 57 cm. All images were presented on a grey background and sized to a width and height of 14.2 × 10.6°. Two images were presented per trial centred 8.1° to the left and right of the vertical meridian and centred on the horizontal meridian. Eye movements of the right eye were recorded using an EyeLink II tracker with a sampling rate of 500 Hz. A chin rest was used, and participants were placed in a set position and requested not to move during the study. A standard 9-point grid was used to calibrate eye movements before each task.

#### Design, Procedure, and Eye-Tracking Analyses

We followed the same procedure as employed in Experiment 1 where participants were assigned to either the stippling (nonartist: *n *=* *16, artist: *n *=* *8); stroking (nonartist: *n *=* *16, artist: *n *=* *8); or control (nonartist: *n *=* *16, artist: *n *=* *8) condition. Twelve artworks were displayed in both the aesthetic rating and eye-tracking tasks, with 24 trials presented in the eye-tracking task (see Figure 5). Eye-tracking analyses were identical to Experiment 1, and ratings of familiarity were also gathered.

### Results

#### Effect of Congruent Action on Aesthetic Ratings

**Figure 6.**
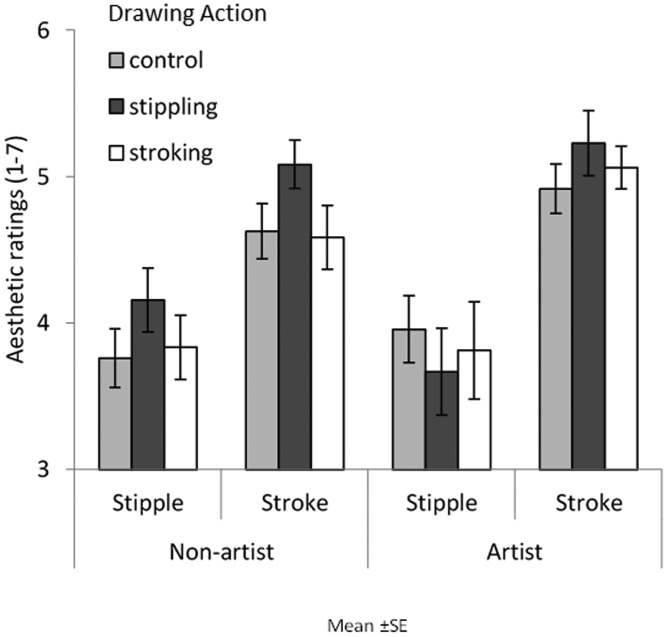
Mean aesthetic ratings of pointillism and brushstroke paintings for both artist and nonartist in the three assigned conditions (control, stippling, and stroking). Error bars show between-group standard error.

Figure 6 shows aesthetic ratings for image style as a function of action conditions and expertise. A three-way ANOVA with image style, action type, and expertise was conducted. A main effect of image style was found as participants aesthetically preferred brushstroke paintings (*M *=* *4.92) to pointillism paintings (*M *=* *3.87), *F*(1,66)= 93.835, *MSE *=* *0.377, *p *<* *.001, ${\text{η}}^{\text{2}}$ = .587. There was no main effect of expertise, *F*(1,66) = 0.397, *MSE *=* *0.818, *p *=* *.531, ${\text{η}}^{\text{2}}$ = .006, and no main effect of action type, *F*(2,66) = 0.804, *MSE *=* *0.818, *p *≤* *.452, ${\text{η}}^{\text{2}}$ = .024. No significant interactions were found between image style and action type, *F*(2, 66) = 0.842, *MSE = *0.377, *p = *.435, ${\text{η}}^{\text{2}}$ = .025; image style and expertise, *F*(1, 66) = 3.558, *MSE = *0.377, *p = *.064, ${\text{η}}^{\text{2}}$ = .051; action type and expertise, *F*(2, 66) = 0.728, *MSE = *0.818, *p *=* *.487, ${\text{η}}^{\text{2}}$ = .022; or image style, action type, and expertise, *F*(2, 66) = 0.561, *MSE = *0.377, *p = *.573, ${\text{η}}^{\text{2}}$ = .017.

#### Effect of Congruent Action on Gaze Behaviour

**Figure 7.**
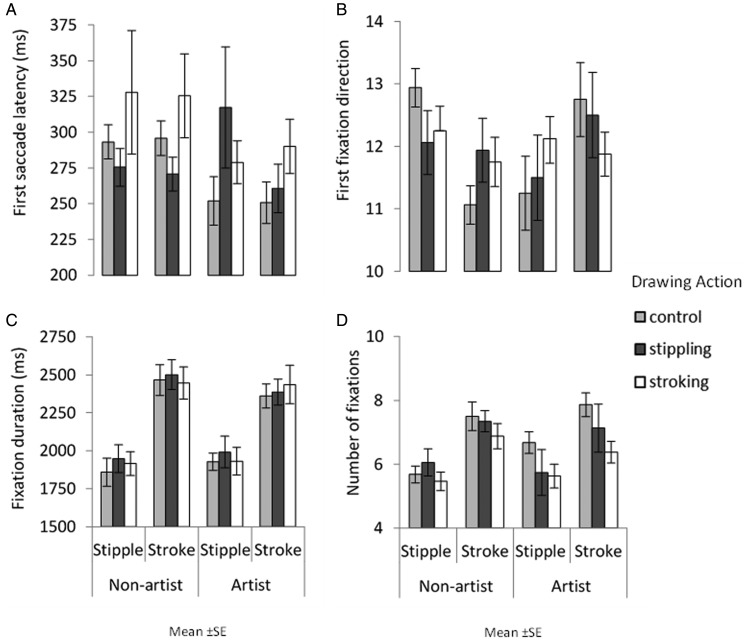
Upper row shows first saccade response: the mean latency of the response in milliseconds (A) and its direction, which is collapsed across expertise and action condition (B). Lower row shows overall fixation behaviour collapsed across condition and expertise: mean total fixation duration in milliseconds (C) and the mean number of fixations (D). Error bars show between-group standard error.

Figure 7 shows gaze behaviour elicited when viewing paintings. A series of separate three-way ANOVAs were conducted for first saccade latency, first fixation direction, fixation duration, and fixation count with image style, action type, and expertise as factors.

No main effect of image style on first saccade latency (Figure 7A) was found, *F*(1, 66) = 1.077, *MSE *=* *2211.235, *p *=* *.303, ${\text{η}}^{\text{2}}$ = .016; no main effect of action type was found, *F*(2, 66) = 1.007, *MSE *=* *12606.53, *p *=* *.371, ${\text{η}}^{\text{2}}$ = .030; and no main effect of expertise was found, *F*(1, 66) = 1.411, *MSE *=* *12606.53, *p *=* *.239, ${\text{η}}^{\text{2}}$ = .021. No significant interactions were found between image style and action type, *F*(2, 66) = 1.762, *MSE = *2211.235, *p = *.180, ${\text{η}}^{\text{2}}$ = .051; image style and expertise, *F*(1, 66) = 0.739, *MSE = *2211.235, *p = *.393, ${\text{η}}^{\text{2}}$ = .011; action type and expertise, *F*(2, 66) = 0.989, *MSE = *12606.53, *p *=* *.377, $\eta^{2}$ = .029; or image style, action type, and expertise, *F*(2, 66) = 1.381, *MSE = *2211.235, *p = *.259, ${\text{η}}^{\text{2}}$ = .040.

No main effect of image style on first fixation direction (Figure 7B) was found, *F*(1, 66) = 0.011, *MSE *=* *5.189, *p *=* *.981, ${\text{η}}^{\text{2}}$ < .001; no main effect of action type was found, *F*(2, 66) < 0.001, *MSE *=* *0.242, *p *=* *1.000, ${\text{η}}^{\text{2}}$ < .001; and no main effect of expertise was found, *F*(1, 66) < 0.001, *MSE *=* *0.242, *p *=* *1.000, ${\text{η}}^{\text{2}}$ < .001. No significant interactions were found between image style and action type, *F*(2, 66) = 0.372, *MSE = *5.189, *p = *.691, ${\text{η}}^{\text{2}}$ = .011; image style and expertise, *F*(1, 66) = 3.865, *MSE = *5.189, *p = *.054, ${\text{η}}^{\text{2}}$ = .055; action type and expertise, *F*(2, 66) < 0.001, *MSE *=* *0.242, *p *=* *1.000, ${\text{η}}^{\text{2}}$ < .001; or image style, action type, and expertise, *F*(2, 66) = 1.336, *MSE = *5.189, *p = *.270, ${\text{η}}^{\text{2}}$ = .039.

For fixation duration (Figure 7C), a main effect of image style was found with all participant’s fixating for longer on brushstroke paintings (*M *=* *2430.21) compared with pointillism paintings (*M *=* *1929.43), *F*(1, 66) = 40.467, *MSE *=* *198311.7, *p *<* *.001, ${\text{η}}^{\text{2}}$ = .380. No main effect of action type was found, *F*(2, 66) = 0.773, *MSE *=* *43331.84, *p *=* *.466 ${\text{η}}^{\text{2}}$ = .023. No main effect of expertise was found, *F*(1, 66) = 0.245, *MSE *=* *43331.84, *p *=* *.623 ${\text{η}}^{\text{2}}$ = .004. No significant interactions were found between image style and action type, *F*(2, 66) = 0.028, *MSE *=* *198311.7, *p = *.972, ${\text{η}}^{\text{2}}$ < .001; image style and expertise, *F*(1, 66) = 0.627, *MSE *=* *198311.7, *p = *.431, ${\text{η}}^{\text{2}}$ = .009; action type and expertise, *F*(2, 66) = 0.092, *MSE *=* *43331.84, *p *=* *.912, ${\text{η}}^{\text{2}}$ = .003; or image style, action type, and expertise, *F*(2, 66) = 0.104, *MSE *=* *198311.7, *p = *.902, ${\text{η}}^{\text{2}}$ = .003.

For the number of fixations made (Figure 7D), a main effect of image style was found with all participant’s making more fixations to brushstroke paintings (*M *=* *7.18) compared with pointillism paintings (*M *=* *5.88), *F*(1, 66) = 41.203, *MSE *=* *1.308, *p *<* *.001, ${\text{η}}^{\text{2}}$ = .384. There was no main effect of action type, *F*(2, 66) = 2.629, *MSE *=* *2.934, *p *=* *.080, ${\text{η}}^{\text{2}}$ = .074, nor was there an effect of expertise, *F*(1, 66) =0.083, *MSE *=* *2.934, *p *=* *.774, ${\text{η}}^{\text{2}}$ < .001. No significant interactions were found between image style and action type, *F*(2, 66)= 0.336, *MSE *=* *1.308, *p = *.716, ${\text{η}}^{\text{2}}$ = .010; image style and expertise, *F*(1, 66)= 1.046, *MSE *=* *1.308, *p = *.310, ${\text{η}}^{\text{2}}$ = .016; action type and expertise, *F*(2, 66) = 1.013, *MSE *=* *2.934, *p *=* *.* *369, ${\text{η}}^{\text{2}}$ = .030; or image style, action type, and expertise, *F*(2, 66) = 0.406, *MSE *=* *1.308, *p = *.668, ${\text{η}}^{\text{2}}$ = .012.

#### Familiarity of Paintings

**Figure 8.**
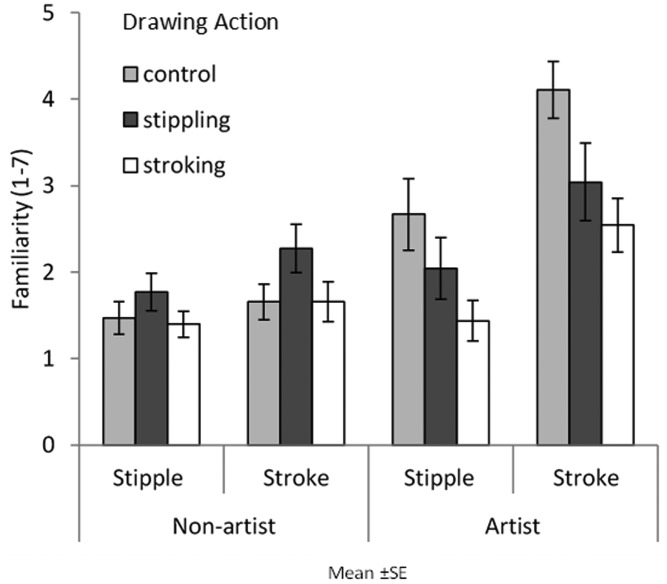
Artists’ and nonartists’ mean familiarity ratings of all pointillism and brushstroke paintings are displayed according to each condition that participants were assigned to (control, stippling, and stroking). Error bars show between-group standard error.

Figure 8 shows artists’ and nonartists’ familiarity ratings for brushstroke and pointillism paintings. A three-way ANOVA was conducted with image style, action type, and expertise as factors.

A main effect of image style was found as participants were more familiar with brushstroke paintings (*M *=* *2.55) than pointillism paintings (*M *=* *1.80), *F*(1,66)= 68.693, *MSE *=* *0.261, *p *<* *.001, ${\text{η}}^{\text{2}}$ = .510. There was a main effect of expertise on familiarity, and artists were more familiar with all artworks (*M *=* *2.64) compared with nonartists (*M *=* *1.70), *F*(1, 66) = 20.101, *MSE *=* *1.394, *p *<* *.001, ${\text{η}}^{\text{2}}$ = .233. There was also a main effect of condition, and those in the control condition (*M *=* *2.47) were more familiar with artworks than those in the stroking condition (*M *=* *1.76), *F*(2, 66) = 4.203, *MSE *=* *1.394, *p *=* *.019, ${\text{η}}^{\text{2}}$ = .113.

An interaction was found between image style and expertise that further explains the main effect of expertise, *F*(1, 66) = 22.927, *MSE *=* *0.261, *p *<* *.001, ${\text{η}}^{\text{2}}$ = .258. Pairwise comparisons reveal that artists (*M *=* *3.23) were more familiar with brushstroke paintings than nonartists (*M *=* *1.86, *p *<* *.001) and that artists (*M *=* *2.05) were more familiar with pointillism paintings than nonartists (*M *=* *1.55, *p *=* *.017). An interaction was found between expertise and condition further explaining the main effect of condition, *F*(2, 66) = 1.394, *MSE *=* *6.305, *p *=* *.014, ${\text{η}}^{\text{2}}$ = .113. Pairwise comparisons reveal that artists in the control condition (*M *=* *3.39) were more familiar with artworks than artists in the stroking condition (*M *=* *1.99, *p *=* *.004). No significant interactions were found between image style and action type, *F*(2, 66) = 0.173, *MSE *=* *0.261, *p *=* *.841, ${\text{η}}^{\text{2}}$ < .001, or image style, action type, and expertise, *F*(2, 66) = 1.441, *MSE *=* *0.261, *p *=* *.244, ${\text{η}}^{\text{2}}$ = .042.

### Discussion

We found no effect of congruency using artworks portraying different artistic techniques (pointillism and brushstroke paintings) and do not support the hypotheses of Experiment 2. Generally, the results of Experiment 2 show brushstroke paintings to be rated as more pleasing than pointillism paintings. This supports McLean et al. (2015) who also found that brushstroke paintings were aesthetically preferred regardless of simultaneous congruent or incongruent actions. In addition, participants fixated for longer and more often on brushstroke paintings and reported these to be more familiar than pointillism paintings. This supports Williams et al. (2018) suggesting that gaze behaviour is driven by aesthetic preferences. Here, we find that coupling the actions of an artist with the actions of a viewer, whether congruent or incongruent, does not impact overall aesthetic ratings or gaze behaviour. Again, both artists and nonartists did not feel that undergoing simultaneous actions influenced their aesthetic ratings. Both Experiments 1 and 2 show some effects of image and action type on aesthetic ratings and gaze behaviour, but none of these are modulated by the congruency of the participant’s actions with artist’s actions.

Ticini et al. (2014) found an effect of congruency on aesthetic judgements when priming participants with hand actions. When primed and later presented with stippling hand actions, ratings for pointillism paintings were higher than when presented with stroking and no hand action primes. Park et al. (2015) suggest that viewers may simulate the movement of the painter once they are more aware of the artist and how they create the art. Albarracín et al. (2008) suggest that priming both perception and action can lead to increases of perceiving, imitating, and adopting the behaviour of others. Experiment 3 adopts a training and priming paradigm to further examine if aesthetic ratings and gaze behaviour is influenced by congruent actions when associations with the actions of the artist are enhanced.

## Experiment 3

One potential explanation for the lack of a congruency effect is the extent to which the link between the participant’s action and that involved in the creation of the artwork is obvious to the participant. To examine this potential explanation, Experiment 3 employed a pretraining phase. Here, associations between art production techniques and the actions produced by participants were linked through visuomotor and visuovisual training allowing participants to gain a greater understanding of the different hand actions made to create artworks (brushstroke and pointillism paintings). A training phase was completed where participants learned painting actions (stippling and stroking) associated with different hand posture images and were primed with these images prior to observing artworks (e.g., pointillism associated with a brush held between the thumb and forefinger for precision grip to induce a stippling motion and brushstrokes associated with coarser force grip).

As with Experiments 1 and 2, aesthetic ratings and gaze behaviour were recorded. As we found no differences of the effects of congruency on the basis of expertise, only nonartists were recruited here. We hypothesise that when a priming hand posture is presented that is congruent to the actions involved in producing the artwork observed, then aesthetic ratings will be greater than when participants are presented with an incongruent or a no action hand prime. Furthermore, we also hypothesise that when a congruent priming hand posture is presented, then gaze (first saccade latency, first fixation, fixation duration, and fixation count) will be influenced and be directed towards the congruent artwork more so than the incongruent artwork. No difference will be found when no action hand grips are presented.

### Method

#### Participants

In this final experiment, 30 participants took part who were not involved in Experiments 1 or 2 (ages 18–42; 27 females, 3 males). The participants had less than 2 years (*M *=* *0.13) of art training and art experience. All participants had normal or corrected-to-normal vision and completed each stage of the study.

#### Apparatus and Materials

**Figure 9.**
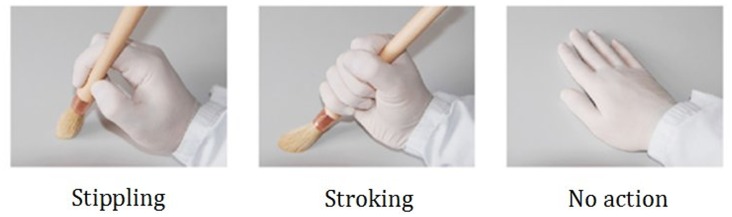
Example of hand posture images (*images adopted from* Ticini et al., 2014).

All apparatus and materials, including paintings, were identical to Experiment 2. Hand posture images (see Figure 9) were sized equally to all artworks. It is worth emphasising that only pointillism paintings were included in Ticini et al.’s (2014) study. To generalise conclusions further, we presented both brushstroke- and pointillism-style paintings.

**Figure 10.**
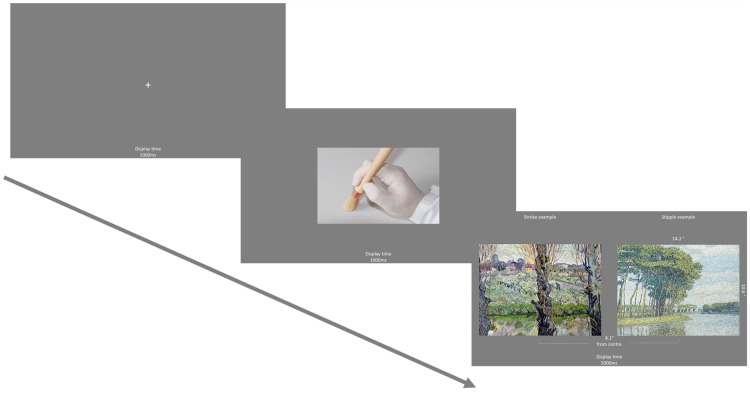
Schematic trial example.

#### Design, Procedure, and Eye-Tracking Analyses

In a training phase, participants were shown three hands that represented three different actions: stippling, stroking, and no action (see Figure 9). The participants adopted the hand shape shown and produced the actions represented 6 times for 10 seconds prior to completing the eye-tracking and pleasingness rating tasks (see explicit instructions later). They held no implement while doing this, simply moving their hand in response to the photograph shown. They were explicitly told to produce the painting movement represented, and their own hands were visible throughout the training phase, thereby allowing links to be formed between the motor output and the photograph.

Training instructions:

- Stroking action: perform actions of drawing lines of approximately 10 cm using the stroking grip.
- Stippling action: perform actions of making dots by tapping at your own pace using the stippling grip.
- No action: place hand palm down on the table.

As with Experiments 1 and 2, participants completed an aesthetic rating task and a free-view eye-tracking task.

##### Aesthetic Rating Task

Participants rated how visually pleasing the 12 paintings were; each painting was rated 3 times (after observing all hand images). All images were presented for 5,000 ms before an aesthetic judgement was made. Prior to this, participants were presented with a hand image for 1,000 ms. This task was completed either before or after the eye-tracking task to control for order effects. A 7-point scale (1: *very displeasing* to 7: *very pleasing*) was used where ratings were made verbally and were recorded by the experimenter.

##### Eye-Tracking Task

For the free-view eye-tracking task, participants were simply informed that they would be presented with pairs of artworks that they could freely view. They completed 24 trials in which they viewed two artworks for 5,000 ms. Prior to each trial, a hand prime image (eight trials for each hand action type) was presented in a randomised order for 1,000 ms. For each trial, a fixation cross was initially displayed for 1,000 ms after which a drift correction was applied. The order of all 24 trials was kept consistent using an originally randomised hand prime order where all images were presented at least once with one of the three hand primes (see Figure 10). Eye-tracking analyses were identical to Experiment 1.

### Results

#### Effect of Congruent Action on Aesthetic Ratings

**Figure 11.**
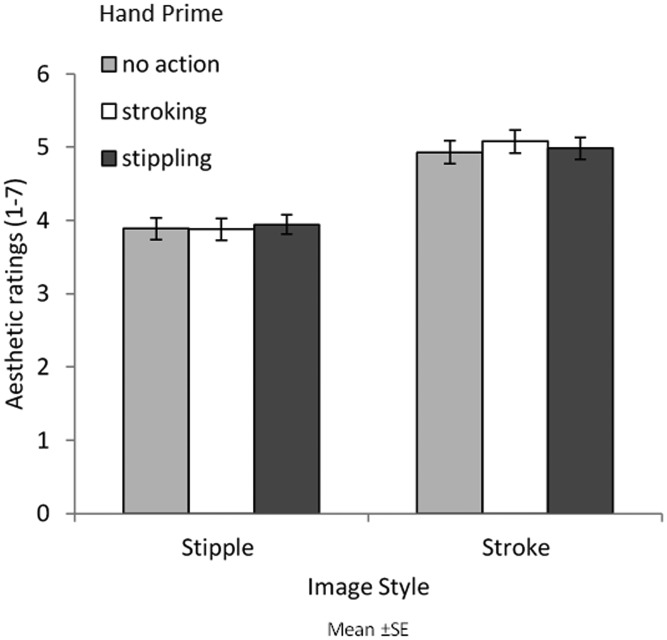
Mean aesthetic ratings of pointillism and brushstroke paintings when presented with each hand prime (no action, stroking, and stippling). Error bars show standard error.

Figure 11 shows aesthetic ratings for image style as a function of hand image prime. A two-way ANOVA with image style and hand prime showed only a main effect of image style on aesthetic ratings as brushstroke paintings (*M *=* *5.00) were aesthetically preferred to pointillism paintings (*M *=* *3.90), *F*(1, 29) = 35.787, *MSE *=* *1.501, *p *<* *.001, ${\text{η}}^{\text{2}}$=.552. No main effect of hand image or interactions were found: *F*(2, 58) = 0.927, *MSE *=* *0.087, *p *=* *.402, ${\text{η}}^{\text{2}}$=.031; *F*(2, 58) = 1.131, *MSE *=* *0.115, *p *=* *.330, ${\text{η}}^{\text{2}}$ = .038, respectively.

#### Effect of Congruent Action on Gaze Behaviour

**Figure 12.**
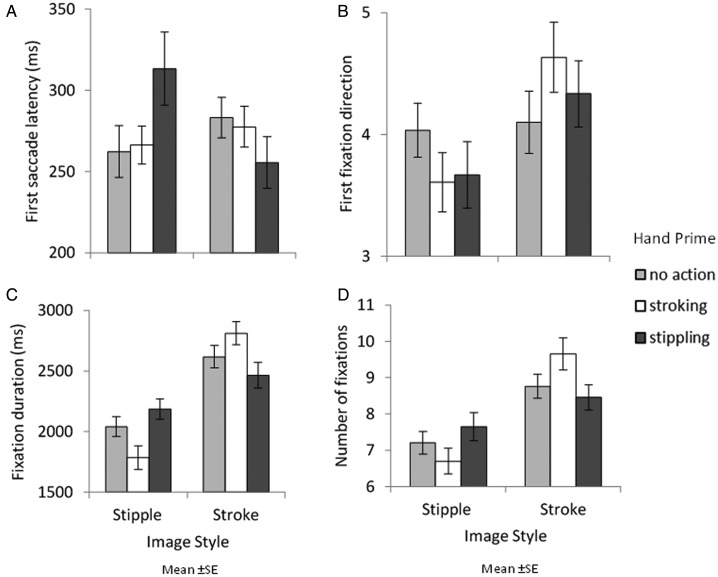
Upper row shows first saccade response: the mean latency of the response in milliseconds (A) and its direction, which is collapsed across hand prime type (B). Lower row shows overall fixation behaviour: mean total fixation duration in milliseconds (C) and the mean number of fixations (D). Error bars show standard error.

Figure 12 shows gaze behaviour elicited when viewing paintings. A series of two-way ANOVAs were carried out examining gaze behaviour with image style and hand image prime as factors.

No main effects of image style or hand image prime were found on first saccade latency (Figure 12A): *F*(1, 29) = 0.782, *MSE *=* *4147.161, *p *=* *.384, ${\text{η}}^{\text{2}}$ = .026; *F*(2, 58) = 0.437, *MSE *=* *6769.689, *p *=* *.648, ${\text{η}}^{\text{2}}$ = .015, respectively. However, an interaction between image style and hand image prime was found, *F*(2, 58) = 7.472, *MSE *=* *3710.153, *p *<* *.001, ${\text{η}}^{\text{2}}$ = .205. Pairwise comparisons showed this was driven by quicker first saccade latencies to brushstroke paintings (*M *=* *255.54) compared with pointillism paintings when preceded by a stipple prime (*M *=* *313.35, *p *=* *.006).

For first fixation direction (Figure 12B), a main effect of image style was found with more first fixations being made to brushstroke paintings (*M *=* *4.36) compared with pointillism paintings (*M *=* *3.64), *F*(1, 29) = 8.398, *MSE *=* *2.710, *p *=* *.007, ${\text{η}}^{\text{2}}$ = .225. No main effects of hand image prime or interactions were found: *F*(2, 58) ≤0.001, *MSE *<* *0.001, *p *=* *1.000, ${\text{η}}^{\text{2}}$<.001; *F*(2, 58) = 0.802, *MSE *=* *5.346, *p *=* *.453, ${\text{η}}^{\text{2}}$ = .027, respectively.

For fixation duration (Figure 12C), a main effect of image style was found as brushstroke paintings were fixated on longer (*M *=* *2631.506) than pointillism paintings (*M *=* *2004.617), *F*(1, 29) = 30.506, *MSE *=* *579715.130, *p *<* *.001, ${\text{η}}^{\text{2}}$ = .513. No main effect of hand image prime was found, *F*(2, 58) = 1.956, *MSE *=* *8518.031, *p *=* *.151, ${\text{η}}^{\text{2}}$ = .063. An interaction between image style and hand image prime was found displaying a congruency effect, *F*(2, 58) = 4.962, *MSE *=* *425933.224, *p *=* *.01, ${\text{η}}^{\text{2}}$ = 0.146. Participants fixated longer on brushstroke paintings when the stroke prime was presented (*M *=* *2811.32) compared with when the stipple prime was presented (*M *=* *2465.28, *p *=* *.022).

A main effect of image style was found for the number of fixations made (Figure 12D) as brushstroke paintings (*M *=* *8.96) were fixated on more so than pointillism paintings (*M *=* *7.19), *F*(1, 29) = 30.843, *MSE *=* *4.582, *p *<* *.001, ${\text{η}}^{\text{2}}$ = .515. No main effect of hand image prime was found, *F*(2, 58) = 1.538, *MSE *=* *0.376, *p *=* *.223, ${\text{η}}^{\text{2}}$ = .050. However, an interaction between image style and hand image prime was found, *F*(2, 58) = 3.821, *MSE *=* *4.672, *p *=* *.028, ${\text{η}}^{\text{2}}$ = .116. Here, an effect of congruency was found but in a different manner; participants did not make significantly more fixations to brushstroke paintings (*M *=* *8.45) compared with pointillism paintings when presented with the stipple hand prime (*M *=* *7.65, *p *=* *.181) but did so when presented with the no action prime (brushstroke paintings; *M *=* *8.76; pointillism paintings, *M *=* *7.20, *p *=* *.002) and the stroke hand prime (brushstroke paintings, *M *=* *9.65; pointillism paintings, *M *=* *6.70, *p *<* *.001).

### Discussion

As with Experiment 2, aesthetic ratings were found to be influenced by image style with brushstroke paintings being aesthetically preferred to pointillism paintings. Gaze behaviour was also affected by style with more first fixations being towards brushstroke paintings. This was followed by more fixations and greater duration throughout the viewing period. We did not find an effect of congruency with aesthetic ratings as found by Ticini et al. (2014); this may be due to differences in the methodology. We did not allow participants to hold a painting instrument or create a painting; thus, our prime may have been less effective. However, we also examined the impact of congruency on brushstroke paintings as well as pointillism paintings and found an effect of congruency on gaze behaviour that supports our hypotheses to some extent. More time was spent fixating on brushstroke paintings when presented with a stroke prime compared with a stipple prime, and a greater number of fixations were not made to brushstroke paintings in comparison with pointillism paintings when preceded by a stipple prime, suggesting an effect of congruency in terms of interference. These results provide some support for a link between the actions involved in the creation of an artwork and gaze behaviour towards congruent artworks. Congruent primes influence gaze behaviour by encouraging greater exploration of preferred artwork styles.

## General Discussion

It has been suggested that while viewing art, perceivers are influenced by the art-making process of the artist (Tinio, 2013). Some recent studies have demonstrated that the actions of the artist can influence aesthetic judgements when observers simultaneously create or familiarise themselves with congruent and incongruent actions (Leder et al., 2012; Ticini et al., 2014), with other studies showing an increase in aesthetic appreciation when the production process is explicitly presented (Chamberlain et al., 2018; McLean et al., 2015). Relationships between the artist and perceiver have been extended to understand eye movement behaviour while viewing art, and it is apparent that gaze is influenced due to the creator of the artwork and having greater knowledge of the creative process (Alvarez et al., 2015; Ishiguro et al., 2016). However, research has not further explored gaze behaviour when a perceiver mimics or is trained in actions that are associated with the creative process of the artist. Here, we examined the impact of congruent actions on aesthetic judgements and gaze behaviour through three experiments that allowed perceivers to resonate with the artist.

In Experiment 1, we found no effect of congruency when geometric stimuli with clear representations of stippling and stroking actions were presented. Actions seem to have little effect on the perceiver, and no differences were found due to artistic expertise. In Experiment 2, using artworks, we continue to find no effect of congruency on aesthetic ratings and gaze behaviour. Brushstroke paintings were more pleasing, more familiar, and fixated on for longer and more often, despite producing congruent, incongruent, or no actions. This supports Williams et al. (2018) who found that eye movements were made towards images rated to be more aesthetically pleasing. The current findings do not support an effect of congruency on aesthetic judgements and gaze behaviour. It may be the case that the top-down processes resulted in no effect of congruency being found. Perceivers continue to fixate on aesthetically pleasing and familiar artworks despite engaging in congruent and incongruent actions with the artist.

In Experiment 3, the inclusion of a training phase allowing participants to be familiar with two distinctive painting actions and priming participants led to a congruent effect being found on gaze behaviour. As in Experiment 2, brushstroke paintings were more pleasing and fixated on longer and more often; here, first fixation was also directed towards brushstroke paintings. However, the presentation of a hand prime influenced gaze such that the brushstroke paintings were fixated on more when presented with a congruent prime than when presented with a stipple prime. Furthermore, modulatory effects of hand primes on number of fixations were also found. A greater number of fixations were not made to brushstroke paintings when stipple primes were presented, despite this being the case for other trial combinations; this suggests an effect of congruency in terms of interference.

Surprisingly, congruent effects were not found with aesthetic judgements throughout all three experiments. Gaze behaviour appears to be driven by overall artwork style preferences (brushstroke paintings) when providing perceivers with an experience of the art-making process. When more explicit methods were used to connect the artist’s actions to the perceivers, then gaze was found to be more towards brushstroke artworks when presented with a congruent prime. A reduction in fixations was found to such preferences when a stipple prime was presented. Differences here with the paradigms used in earlier experiments can be evaluated as we consider an explanation for our congruency effects. These are only found with gaze behaviour once hand primes of painting techniques and a training phase are introduced. This allows for visual feedback of participant’s hand gestures and experience to both stroking and stippling actions.

One explanation for our results may lie in suggested links between art and embodiment as subserved by a mirror neuron system (Freedberg & Gallese, 2007). Mirror neurons are activated both when an action is performed by the person and when the same action is performed by another individual. This interaction leads to the fluency between action and observation, increasing preference. The way in which art is produced and the actions of the artist is suggested to impact the aesthetic experience of the perceiver due to activation of these embodied mechanisms. (Freedberg & Gallese, 2007; Rizzolatti & Craighero, 2004). Piechowski-Jozwiak et al. (2017) state how the visible marks from creation and imagining the actions and directions of the artist’s movement activates areas in the perceiver’s brain that leads to feeling a similar experience to the artist. Experimental studies show how participants perceive more movements in real artworks in comparison with modified computerised versions. Observations of real artworks produced greater cortical activation and higher aesthetic ratings were given to these original works. It is suggested that the dynamics of the artworks where the brushstrokes made by the artist can be perceived affected these results (Sbriscia-Fioretti et al., 2013; Umiltà et al., 2012). Furthermore, it has been found that aesthetic ratings positively correlate with perceptions of movement within an artwork and consequential eye movement behaviour (Brinkmann et al., 2019; Cattaneo et al., 2017; Mastandrea & Umiltà, 2016). From the current studies, we do not find support for congruent actions influencing aesthetic judgements, and it is difficult to conclude that gaze behaviour using the training and priming paradigm in Experiment 3 is affected by congruency due this mirror neuron hypothesis.

Another possible explanation of our results is that gaze is influenced by congruency more simply due to fluency, that is, an ease of processing (Leder, 2013). The use of a training phase and hand primes in Experiment 3 may have led to increasing the ease by which artworks are processed. Participants may have an implicit knowledge of the hand actions required to produce observed paintings. Ishiguro et al. (2016) found that providing knowledge about the creative process influenced gaze and specifically found greater exploration with photos that contained recognisable features. The perceptual fluency theory explains that greater fluency through the ease of identifying or relating with a stimulus leads to the stimulus being perceived as more pleasing (Reber et al., 2004). Therefore, this may extend to our gaze behaviour results that show fixation to brushstroke paintings to be influenced by congruent and incongruent primes. Nevertheless, in Experiments 2 and 3, we generally find brushstroke paintings to be preferred, fixated on longer and more often but also indicated to be more familiar. The overall preference for such styles further supports our results to be explained by a greater ease of processing artworks that are more familiar, and we should acknowledge that this difference in familiarity between artwork styles may have led to a lack of congruency effects. Although the distinct action process for creating pointillism and brushstroke paintings makes such stimuli ideal for this area of research, use of additional styles should be considered in future studies to remove this potential issue of familiarity.

In conclusion, our results demonstrate that gaze behaviour is influenced by artwork style preferences even when engaging with the creative processes of artists in the form of producing congruent and incongruent actions. When greater explicit links are made using training methods and hand primes that resonate with the artist’s actions, then congruent and incongruent actions influence gaze behaviour by encouraging greater exploration of preferred artwork styles.

## Supplemental Material

IPE911059 Supplemental Material - Supplemental material for Enhanced Associations With Actions of the Artist Influence Gaze Behaviour Click here for additional data file. Supplemental material, IPE911059 Supplemental Material for Enhanced Associations With Actions of the Artist Influence Gaze Behaviour by Louis Williams, Eugene McSorley and Rachel McCloy in i-Perception
