# Supplementary material for: Enhanced Associations With Actions of the Artist Influence Gaze Behaviour
Source: Iperception. 2020 Mar 12;11(2):2041669520911059. doi: 10.1177/2041669520911059 (PMC7074529; doi:10.1177/2041669520911059)
Supplement: IPE911059 Supplemental Material - Supplemental material for Enhanced Associations With Actions of the Artist Influence Gaze Behaviour [file IPE911059_Supplemental_Material.pdf]

Supplemental materials

A list of the paintings presented in Experiments 2 and 3 is provided below:

Pointillism paintings

1. Seurat, Georges (1859-1891). Port-en-Bessin, cranes and breakthrough
2. Seurat, Georges (1859-1891). Bessin harbor entrance
3. Seurat, Georges (1886). La Maria, Honfleur
4. Baum, Paul (1859-1932). Meadows at the creek
5. Baum, Paul (1904).Trees by a canal
6. Baum, Paul (1859-1932). A view of Sluis in the morning sun

Brushstroke paintings

1. van Gogh, Vincent (1889). View on Arles
2. van Gogh, Vincent (1989). Olive grove
3. Monet, Claude (1840-1926). On the cliffs of Pourville
4. Monet, Claude (1882). The sea at Pourville
5. van Gogh, Vincent (1888). The sea at Saintes Maries
6. van Gogh, Vincent (1888). Coals towboats
